# Acute Effects of Carbohydrate Supplementation on Intermittent Sports Performance

**DOI:** 10.3390/nu7075249

**Published:** 2015-07-14

**Authors:** Lindsay B. Baker, Ian Rollo, Kimberly W. Stein, Asker E. Jeukendrup

**Affiliations:** 1 Gatorade Sports Science Institute, Barrington, IL 60010, USA; E-Mail: kimberly.stein@pepsico.com; 2 Gatorade Sports Science Institute, Beaumont Park, Leicester LE3 9QH, UK; E-Mail: ian.rollo@pepsico.com; 3 School of Sport, Exercise and Health Sciences, Loughborough University, Leicestershire LE11 3TU, UK; E-Mail: jeukendrup@me.com

**Keywords:** glucose, glycogen, intermittent exercise capacity, skill, sprinting, team sports

## Abstract

Intermittent sports (e.g., team sports) are diverse in their rules and regulations but similar in the pattern of play; that is, intermittent high-intensity movements and the execution of sport-specific skills over a prolonged period of time (~1–2 h). Performance during intermittent sports is dependent upon a combination of anaerobic and aerobic energy systems, both of which rely on muscle glycogen and/or blood glucose as an important substrate for energy production. The aims of this paper are to review: (1) potential biological mechanisms by which carbohydrate may impact intermittent sport performance; (2) the acute effects of carbohydrate ingestion on intermittent sport performance, including intermittent high-intensity exercise capacity, sprinting, jumping, skill, change of direction speed, and cognition; and (3) what recommendations can be derived for carbohydrate intake before/during exercise in intermittent sports based on the available evidence. The most researched intermittent sport is soccer but some sport-specific studies have also been conducted in other sports (e.g., rugby, field hockey, basketball, American football, and racquet sports). Carbohydrate ingestion before/during exercise has been shown in most studies to enhance intermittent high-intensity exercise capacity. However, studies have shown mixed results with regards to the acute effects of carbohydrate intake on sprinting, jumping, skill, change of direction speed, and cognition. In most of these studies the amount of carbohydrate consumed was ~30–60 g/h in the form of a 6%–7% carbohydrate solution comprised of sucrose, glucose, and/or maltodextrin. The magnitude of the impact that carbohydrate ingestion has on intermittent sport performance is likely dependent on the carbohydrate status of the individual; that is, carbohydrate ingestion has the greatest impact on performance under circumstances eliciting fatigue and/or hypoglycemia. Accordingly, carbohydrate ingestion before and during a game seems to have the greatest impact on intermittent sports performance towards the end of the game.

## 1. Introduction

Intermittent sports (e.g., team sports) are characterized by intermittent bursts of high-intensity exercise and require the execution of complex sport-specific skills and cognitive tasks over a prolonged period of time (~1–2 h), with longer breaks at scheduled intervals (e.g., quarters, half time) as well as unscheduled times (e.g., injury or restarting play after scoring in soccer or rugby). Performance of intermittent sports is dependent upon a combination of anaerobic and aerobic energy systems, both of which rely on carbohydrate as an important fuel source [1]. Current guidelines for athletes competing in intermittent sports recommend ingesting carbohydrate at a rate of 30–60 g/h during >1 h of training and competition [1,2,3,4]. However, compared with endurance sports, relatively few studies have tested the impact of carbohydrate supplementation during practice or competition on intermittent sport performance. In addition, the complex nature of intermittent sports makes it difficult to accurately and reliably measure performance and the many methodological differences between studies confound the ability to interpret results. Previous reviews of the effects of carbohydrate ingestion on intermittent sport performance have focused on a single sport or category of intermittent sport or discussed only certain aspects of performance [5,6,7]. In this paper we discuss the effect of carbohydrate ingestion on individual performance in intermittent sports, with the specific objectives of reviewing: (1) potential biological mechanisms by which carbohydrate impacts intermittent sport performance; (2) the effect of carbohydrate ingestion on several aspects of intermittent sport performance, including intermittent high-intensity exercise capacity, sprinting, jumping, skill, change of direction speed, and cognition; and (3) what recommendations can be derived for carbohydrate intake before/during exercise in intermittent sports based on the available evidence. 

## 2. Search and Selection Criteria

This narrative review primarily discusses the acute effects of carbohydrate feedings during exercise. Studies in which carbohydrate is ingested shortly prior to, as well as during, exercise are included in this discussion. This is because the carbohydrate ingested in the hour before exercise is likely to contribute to energy provision during exercise. The combined effect of carbohydrate with other nutrients (e.g., protein) or compounds (e.g., caffeine) is not discussed. However, if these studies included a comparison of carbohydrate alone versus placebo those results are discussed here. Additionally, excluded from this review are studies without a placebo arm.

Our aim is to primarily focus on performance outcomes, from studies which protocols mimicked the physical and/or cognitive demand of intermittent sports and utilized intermittent sport athletes as participants. This review includes all intermittent sports that have been studied in this regard, including soccer (football), American football, basketball, rugby, field hockey, and racquet sports (e.g., tennis, squash, *etc.*). Some studies have employed intermittent high-intensity protocols with a mix of intermittent sport athletes, such as soccer, rugby, and/or field hockey. The majority of research in this area has been conducted in soccer. Because the activity pattern of soccer is similar to other intermittent sports, it is perhaps reasonable to extrapolate results from soccer to these other sports. However, it is also important to recognize that although similar in sprint and skill requirements, other sports such as tennis can have quite unique activity durations. Thus, studies applicable to these unique demands will also be discussed. Sports that require skill but do not rely heavily upon intermittent bouts of high-intensity running (e.g., golf, bowling) are not discussed here. We will discuss all performance outcomes that have been researched in relation to carbohydrate intake by intermittent sport athletes, including intermittent high-intensity exercise capacity, sprinting, jumping, sport-specific skills, change of direction speed, and cognitive function.

## 3. Intermittent Sports Overview

The diversity of intermittent sports reflects the array of traditions, heritage, and cultures from which they have evolved [8,9]. Although regional differences exist, the intermittent or “team sport” category is likely the most popular with regard to both participation and spectator interest across the world. A common characteristic of intermittent sports is the pattern of play. All intermittent sports require brief high-intensity exercise such as jumping for a rebound in basketball, a sprint for the ball against an opponent in soccer, or a tackle in American football. Many intermittent sports also require the execution of sport-specific skills, such as a serve in tennis or dribbling, passing, and shooting in soccer or field hockey. The ability to sustain skill execution during a game has clear performance implications. For example, soccer teams with the smallest decrease in skill performance over a match have been found to finish the season in a higher league position [10]. In addition, success in intermittent sports is partially dependent upon attention, decision-making, response time, and other aspects of cognitive function.

Another demand of intermittent sports is agility, which has been identified as a key attribute that may discriminate between elite and sub-elite groups of adolescent soccer players [11]. Although it is recognized within the sports science community as an important component of performance, a clear definition of agility does not exist [12]. It has been proposed, however, that the term agility describes the ability to produce rapid whole-body movement with change of velocity or direction in response to a stimulus. By contrast changes in direction that involve no decision making or reactive component are better described as change of direction speed [12,13]. Most studies that have administered “agility” tests in intermittent sports protocols have measured change of direction speed according to the aforementioned definitions.

In general, team success is dependent upon the cooperation of individual players to score more goals/points than the opposition. However, intermittent sports vary in several ways, including the rules of play (e.g., regarding rest periods and player substitution), size of the playing field, duration between high-intensity efforts, overall duration of the contest, total distance covered, frequency of games, and position-specific requirements. For example, some intermittent sports, such as soccer, are primarily aerobic in nature, as indicated by the large distances covered during competition (~10 km) [14,15]. In this regard, intermittent sports have been categorized into endurance-based (e.g., soccer, field hockey, lacrosse), court (e.g., basketball, volleyball, tennis), batting (e.g., baseball, softball, cricket), and strength and power-based (e.g., American football, rugby) sports [1] (see Table 1). Many aspects of these various intermittent sports, including the high-intensity efforts, aerobic activity, and cognitive demands related to skill, attention, and decision making, are dependent upon carbohydrate as an important substrate for energy production. In the next section, we will discuss the potential biological mechanisms by which carbohydrate may impact intermittent sport performance, including both metabolic and non-metabolic (central) effects.

**Table 1:** Intermittent sport classifications.

Classification	Examples	Sport Distinctions
Endurance-based field sports	Soccer, field hockey, lacrosse	Large playing area, longer distances covered, continuous activity at variable speeds.
Court sports	Basketball, volleyball, tennis	Smaller playing area, shorter duration games, frequent substitution, often several games per day or over several days.
Strength and power field sports	American football, rugby	Shorter distance covered, frequent short bursts, high contact.
Batting field sports	Baseball, softball, cricket	Lower overall energy demands, many hours on field, greater rest duration between efforts.

Table information adapted from Holway and Spriet [1].

## 4. Potential Mechanisms for Effects of Carbohydrate Ingestion on Intermittent Sports Performance

### 4.1. Historical Perspective

As early as 1925, Gordon *et al.* [16] reported that ingestion of candy by runners during a marathon prevented hypoglycemia and improved race times compared with when no candy (sugar) was consumed. In intermittent sports, similar work was pioneered by Cade *et al.* in the early 1970’s [17,18]. In 1971, Cade and colleagues reported the effects of exercise on blood glucose changes in four players of the University of Florida football team during a vigorous 2 h practice session with no food or fluid intake [17]. The football players’ blood glucose concentration decreased progressively throughout practice [18]. This work was followed by a study in 1972 to determine whether carbohydrate replacement could prevent the disturbances in blood glucose concentration [18]. Cade *et al.* [18] found that performance during a standardized walk-run test (7 mile course) was significantly improved when ~1 L of a 3% glucose-electrolyte solution was consumed compared with when the athletes drank the same volume of water. Whereas subjects’ blood glucose concentration decreased during the water intake trials (by 1.3 mmol/L), it increased (by 1.0 mmol/L) while drinking the 3% glucose-electrolyte solution. 

Around the same time, the importance of muscle glycogen to soccer performance was also being investigated in Europe [19,20]. By using the muscle biopsy technique, Agnevik [20] reported that male soccer players were nearly emptied of muscle glycogen after a match and that the greatest rate of glycogen depletion occurred in the first half of the match. In a similar study, muscle samples were biopsied from the quadriceps femoris of recreational players at the beginning, halftime, and end of a soccer match [19]. Saltin [19] reported that muscle glycogen concentrations were significantly lower on completion of the match (pre: 96 mmol/kg wet weight (w.w.); halftime: 32 mmol/kg w.w.; end: 9 mmol/kg w.w.). Those players who began the match with low muscle glycogen (45 mmol/kg w.w.) had almost depleted stores by halftime. This was the first study to report the performance implication associated with muscle glycogen concentrations. Specifically, players who began the match with high muscle glycogen covered a greater distance and spent more of the total time completing high-intensity runs compared with those players who began the game with low muscle glycogen (24% *vs.* 15% of the match time in high *vs.* low muscle glycogen players, respectively). These findings corresponded with studies analyzing the activity profile of professional soccer players in the UK [21], which reported that players covered less distance in the second half compared with the first half of a match. Since these early carbohydrate studies in American football and soccer there has been continued research exploring carbohydrate ingestion and intermittent sports performance. 

### 4.2. Metabolic Effects of Carbohydrate

#### 4.2.1. Carbohydrate, Exercise Metabolism, and Fatigue

Fundamentally, the deterioration of running performance during intermittent sports is a consequence of fatigue. Due to the physiological demands of intermittent sports, fatigue manifests at different times during a contest and can be a consequence of distinctly different mechanisms. For example, players experience temporary fatigue following the most intense periods throughout a game and more permanent fatigue in the final phases of a game [22,23]. The transient fatigue that players experience during the most intense periods of intermittent activity is associated with repeated efforts combined with an insufficient recovery time (typically <30 s). The factors that affect recovery time and subsequent running performance during competition are complex and include tactical and pacing strategies; we direct readers towards the commentary by Paul *et al.* [24] on this topic. Nevertheless, under these circumstances, a high anaerobic energy turnover results in an intramuscular accumulation of hydrogen ions and inorganic phosphate as well as the depolarization of the resting membrane potential. The depolarization of the resting membrane potential has been reported to be a result of disturbances in the muscle sodium, potassium, and chloride homeostasis [22,25]. The precise mechanisms underpinning transient fatigue are beyond the scope of this review but are unlikely to be influenced by carbohydrate provision as long as glycogen concentrations remain above a certain critical level (~200 mmol/kg dry weight (d.w.)) in active skeletal muscle [26]. 

#### 4.2.2. The Role of Muscle Glycogen

It is well established that carbohydrate and fat are the two primary fuel sources oxidized by skeletal muscle tissue during prolonged (endurance-type) exercise. The relative contribution of these fuel sources largely depends on the exercise intensity and duration, with a greater absolute and relative contribution from carbohydrate as exercise intensity increases above ~60% VO_2_max. Muscle glycogen provides a rapidly available substrate for energy production when completing high intensity efforts during intermittent activity. For example, in a single 6-sec sprint muscle glycogen contributes ~50% to adenosine triphosphate (ATP) turnover within the muscle [27,28]. Thus, the consequence of repeated sprint activity is a net reduction in muscle glycogen concentrations [29]. Although glycogen is depleted in type I and II muscle fiber types, it may be the specific depletion of glycogen in type II muscle fibers that results in the significant loss in power output during repetitive sprints [30]. It may be that the decrease in muscle glycogen below a critical level in response to variable intensity running contributes to the more permanent fatigue experienced towards the end of a game [31]. Furthermore, the impact of low muscle glycogen is likely to have a greater consequence in sports where the endogenous store of glycogen is insufficient to meet the energy demands over the duration of the exercise (e.g., extra time or over time in tournament competition). 

In one study (employing the Loughborough intermittent shuttle running test (LIST); a protocol mimicking the demands of soccer and similar team sports [32]), muscle biopsy analysis revealed a significant reduction in muscle glycogen concentration in type I and II muscle fibers from before to after exercise. However, muscle glycogen use was reduced by 22% when players ingested a 6.9% carbohydrate solution throughout exercise compared with placebo [33]. The preservation of muscle glycogen is a viable mechanism to explain why players consuming carbohydrate are able to sustain high-intensity running in the second half of live soccer matches. For example, the performances of ten soccer players were video-recorded on two separate occasions: when players drank either 400 mL of a concentrated carbohydrate solution (16% maltodextrin) or placebo before and during halftime of the match. The players who drank the carbohydrate solution ran ~40% greater distance during the second half of the game, in comparison with when the placebo beverage was consumed [34]. An important consideration when interpreting performance data during any team sport is the high variability observed between games, as the tactical formation and level of competition have been reported to influence the distance a player covers at high speed [35]. Thus, although an interesting measure, assessing the impact that carbohydrate ingestion has on team sport performance in live matches is challenging due to the complex interaction between physical and technical components.

A study employing the Copenhagen Soccer Test (CST) [36] obtained frequent and rapid measurements of muscle and blood metabolites allowing insight regarding the anaerobic energy turnover and rates of muscle glycogen use in various phases of a 90-min simulated soccer match [36]. Type I and type II muscle fibers exhibited significant glycogen depletion, with ~80% of fibers being depleted or almost depleted (<200 mmol/kg d.w) of glycogen after 90 min of intermittent activity. Muscle glycogen concentrations < ~200 mmol/kg d.w have been shown to significantly decrease the glycolytic rate [37]. In addition, the depletion of muscle glycogen in sub-cellular glycogen compartments (*i.e.*, sarcoplasmic reticulum) results in concomitant reductions in muscle calcium handling [38]. A reduced rate of sarcoplasmic reticulum vesicle calcium release has been reported to reduce peak power output [39]. Thus, low muscle glycogen influences the flux of calcium and impairs the contractile property of the muscle. 

The rate of muscle glycogen utilization has been found to decrease from the first to second half of a simulated soccer match. Specifically, in a study employing the CST, the rate of muscle glycogen use was reported to be highest (4.0 ± 1.2 mmol/kg d.w./min) during the warm-up and the first 15 min of simulated play. By comparison, muscle glycogen use was significantly lower from 15 to 60 min (1.8 ± 0.5 mmol/kg d.w./min) and lower still from 60 to 90 min (0.9 ± 1.2 mmol/kg d.w./min) [36]. In a study utilizing the LIST protocol, participants ingested either a 6.4% carbohydrate-electrolyte solution (~90 g/h) or a placebo immediately before and at 15-min intervals during exercise. A similar rate of muscle glycogen use from before exercise to 90 min (~2 mmol/kg d.w./min) was reported in the carbohydrate and placebo trials [40]. In this study participants continued to complete blocks of the LIST after 90 min to volitional fatigue. All participants ran longer during the carbohydrate trial (158.0 ± 28.4 min) compared with the placebo trial (131.0 ± 19.7 min), representing a 21% increase in intermittent running capacity. In contrast to previous studies [33], fatigue occurred at similar muscle glycogen concentrations in both trials (~200 mmol/kg d.w). Concentrations of plasma glucose and serum insulin were higher in the carbohydrate trial than the placebo trial at the point of fatigue, suggesting a role of greater glucose availability in the superior performance.

#### 4.2.3. The Role of Blood Glucose

The metabolic response to carbohydrate ingestion differs depending whether the individual is at rest or exercising [41]. At rest the response to elevated blood glucose is an upregulation in the synthesis and secretion of insulin within the beta cells of the islets of Langerhans. Insulin causes decreased lipolysis and increased glucose uptake in liver, skeletal muscle, and fat cells [41]. The role of liver glycogen is the regulation of blood glucose concentration (euglycemia: 4.0–5.5 mmol/L). At the onset of exercise, muscular contraction causes an increased uptake of glucose from the blood. In opposition to the effect of insulin, liver glycogenolysis is activated by the actions of glucagon and epinephrine. Russell *et al.* [41] reports that the insulin response to carbohydrate ingestion during the warm up period prior to team sport activity is inhibited by the actions of epinephrine, which accounts for the elevated blood glucose concentrations typically observed during this stage of exercise. It has long been established that as exercise duration increases blood glucose has an increasing contribution to carbohydrate oxidation in the muscle [42]. 

Blood glucose concentration can increase in response to intermittent sport activity due to an increase in circulating catecholamines [43,44,45,46]. Although glucagon is reported to be relatively unchanged during a soccer match, concentrations of epinephrine and norepinephrine increase through the stimulation of the sympathetic nervous system [43,45,46]. Epinephrine stimulates glycogenolysis in the liver which results in an increase in blood glucose concentration above resting values [43]. Although transient decreases in blood glucose concentration have been reported following half time of a soccer match [47,48,49], hypoglycemia is relatively rare during intermittent sports lasting ≤90 min in fed individuals [29,44], suggesting that liver glycogen is sufficient to maintain or even increase blood glucose concentration during a match [29,37,50]. More recently evidence suggests that blood glucose concentration can be well maintained beyond 90 min, even for the duration of extra time (+30 min) in soccer [51]. Nevertheless, team sport players are advised to ingest carbohydrate during exercise for the aforementioned benefits to preserving endogenous glycogen and ability to maintain high intensity running performance late in exercise [52].

An effect of ingesting carbohydrate-electrolyte beverages during intermittent exercise is an increase in blood glucose concentrations during exercise, compared with the ingestion of non-caloric beverages [47,50,51,53]. Although mechanisms remain unclear, authors have suggested that that decision-making and successful skill execution during a soccer match may be influenced by blood glucose concentrations [6]. Elevated blood glucose has been associated with an overall improvement in the percentage of successful serves and return-of-serve success during tennis [54] and skill performance in soccer [55] and basketball [56]. In addition, Bandelow *et al.* [57] showed that high plasma glucose concentration from sports drink ingestion during a soccer match was related to faster response speeds during several cognitive/motor skill tests, including fine motor skill, complex visual discrimination, working memory scanning, and psychomotor skill, following the soccer match. However, it is important to note that in this study the faster response speed in working memory came at the expense of reduced accuracy, so this may have simply been an artifact of a speed/accuracy “trade off”. Blood glucose concentration may influence skill performance since the brain is almost entirely dependent on a continuous supply of glucose from the circulation for optimal functioning [58]. Elevations in blood glucose have been reported to increase the supply of glucose to the brain and preserve the integrity of the central nervous system [58]. Furthermore, elevated blood glucose concentrations have also been associated with muscle glycogen sparing [59], improved neuromuscular function [60] and reduced central fatigue [61]. 

A recent study reported that both a 9.6% carbohydrate solution (plus carbohydrate gel, 142 g carbohydrate/h overall) and a 5.6% carbohydrate solution (plus placebo gel, 54 g carbohydrate/h) before and at half time increased blood glucose concentrations compared with the ingestion of a placebo during a protocol which simulates soccer match play [47]. Mean sprint speed was consistently faster in both the carbohydrate trials (9.6% solution: 5.73 m/s; 5.6% solution: 5.66 m/s) compared with placebo (5.58 m/s) from the start to end of 90 min [47]. It is important to note, that it is not possible to distinguish if the improved performance was due to a dose dependent effect of carbohydrate ingestion on blood glucose concentration, as the participants also ingested caffeine (∼6 mg/kg body mass) with the 9.6% carbohydrate solution. A study by the same research group [51] investigated the impact of carbohydrate (0.7 g/kg body mass) or placebo ingestion on physical and skill performance in the extra time period of a simulated soccer protocol. In this study, carbohydrate was provided in the form of glucose and maltodextrin gels before exercise, half time, and at 90 min. The carbohydrate trial increased blood glucose concentrations and was associated with improved dribbling precision in the extra time period (90–120 min). However, the elevated blood glucose was not able to attenuate the reduction in sprinting and jumping performance observed in this time [51]. When carbohydrate stores are severely reduced during the latter stages of prolonged exercise, the threat to cerebral metabolism may be prevented by discontinuing exercise [40]. It is likely that during exhaustive, high-intensity, intermittent exercise, decreased performance and ultimately volitional fatigue is a multifaceted consequence of peripheral as well as central mechanisms [40,62].

Nybo [60] demonstrated that when endurance-trained cyclists developed hypoglycemia, neuromuscular performance (sustained maximal voluntary contraction) was impaired. The lower force production was reported to be a consequence of central fatigue (*i.e.*, a diminished activation drive from the central nervous system) [60]. When glucose was fed to cyclists to preserve blood glucose concentrations throughout exercise, neuromuscular performance was maintained. The mechanism underlying the hypoglycemia-induced central fatigue has been speculated to be directly related to a reduced delivery of glucose as a substrate to the brain [60]. With regard to intermittent sports, large regions of the brain such as the motor cortex will be activated, as well as regions involved in cardiorespiratory regulation. Endothelial glucose transport may become rate limiting for the cerebral metabolic rate of glucose when the concentrations of arterial glucose fall to hypoglycemic levels [60]. 

Interestingly, transient changes in blood glucose concentrations have been reported during intermittent high-intensity exercise. Specifically, elevated blood glucose concentrations observed during the first half of soccer-specific exercise are negated in the early stages of the second half, when replicating carbohydrate ingestion and passive half-time practices typical of soccer [47,48,49]. This observation is of relevance because physical performance has been reported to be reduced in the early stages of the second half compared with the opening stages of a match [22]. Russell *et al.* [48] reported that blood glucose values recorded at the beginning of the half time period dropped 30% by the beginning of the second half period. In this and other studies which have observed a transient fall in blood glucose over the half time period in response to carbohydrate feedings, blood glucose concentrations are typically similar to those observed in the placebo trials. Importantly, consistent with reports from endurance exercise [63] the transient drop in blood glucose following the half time period has not been associated with decrements in soccer performance [47,48]. Nevertheless, future studies could explore how the magnitude of change in blood glucose concentrations may influence other aspects of skill performance following the half time period. 

Further research is required to establish the precise mechanisms by which blood glucose concentration may impact physical performance, cognitive function, and technical skill. Nevertheless, it is reasonable to conclude that elevated blood glucose is associated with superior skill performance, whilst the maintenance of blood glucose concentrations would improve skill and running performance under circumstances of fatigue and/or hypoglycemia.

### 4.3. Non-Metabolic Effects of Carbohydrate

There is accumulating evidence that carbohydrate ingestion may have a “non-metabolic” central effect. Studies in running and cycling have reported a benefit of routinely mouth rinsing and expectorating a carbohydrate solution on high intensity endurance performance lasting ~30–70 min [64]. Thus, the ergogenic effect of carbohydrate ingestion when the exercise is of high intensity (>75% VO_2_max) and relatively short duration may be mediated via the activation of brain pathways associated with reward and motivation, in response to carbohydrate recognition in the mouth [64]. 

Rauch and colleagues [65] coined the term “glycostat” which describes the process of chemoreceptors monitoring the carbohydrate status of the muscle during exercise and relating that information to the brain. Speculatively, the brain may also monitor incoming energy (*i.e.*, carbohydrate in the mouth), and adjust energy expenditure accordingly. This is based on the observations that when carbohydrate is ingested, reward centers in the brain are activated and corticomotor pathways are excited, before carbohydrate enters the peripheral circulation [66,67]. These studies may also in part explain reported performance enhancements early in exercise with carbohydrate ingestion in fed individuals where a clear metabolic benefit of carbohydrate ingestion is absent [47].

To date, the benefits of mouth rinsing carbohydrate on repeated sprint performance have been investigated by two studies. Dorling and Earnest [68] found no effect of mouth rinsing a 6.4 % maltodextrin solution on the average or fastest time to complete 3 repeated sprint ability tests during the LIST protocol. However, this performance measure during the LIST protocol, in which the subjects were not able to self-pace, may not have been sufficiently sensitive to detect a potential influence of carbohydrate mouth rinse. By contrast, Rollo and colleagues [69] utilized a recently validated self-selected pacing LIST protocol [70]. In this study, mouth rinsing a 10% maltodextrin solution was associated with increased self-selected jogging speed and also an 86% likelihood of benefiting 15 m sprint performance during the final stages (75–90 min) of exercise, in comparison with mouth rinsing a placebo. 

Finally, Turner *et al.* [71] recently demonstrated that a carbohydrate mouth rinse can increase activation within the primary sensorimotor cortex during physical activity (hand-grip motor task) and enhance activation of neural networks involved in sensory (including visual) perception. Whether mouth rinsing carbohydrate is sufficient to improve skill performance is an interesting question. Although specific research examining the effect of carbohydrate mouth rinse on skill performance is currently lacking, the relevance of the results is questionable due to the aforementioned benefit of carbohydrate ingestion on endogenous glycogen and blood glucose.

### 4.4. Summary

In summary, the mechanisms by which carbohydrate ingestion before and during prolonged intermittent exercise effect performance are complex. The importance of carbohydrate availability for intermittent sport activity is evident by studies which have reported severe degradation of endogenous stores during exercise of this nature. The associated fatigue observed with low muscle glycogen translates directly to reduced performance, which may manifest in less high intensity running. Carbohydrate ingestion prior to exercise blunts the hepatic release of glucose and thus preserves the limited endogenous glycogen stores. The magnitude of the impact that carbohydrate ingestion has on intermittent sport performance is likely to be dependent on the carbohydrate status of the individual; that is, carbohydrate ingestion has the greatest impact on performance under circumstances eliciting permanent fatigue and/or hypoglycemia. Finally, further research is required to determine the relative contribution that carbohydrate ingestion may have on metabolic and non-metabolic mechanisms which influence performance.

## 5. Intermittent Sport Performance Research Review

### 5.1. Intermittent High-Intensity Exercise Capacity

For endurance, court, and field and power-based intermittent sports, the capacity to sustain high intensity efforts alternated with rest or lower intensity periods over the duration of a game is critical to the success of an individual athlete. In 2011, Phillips *et al.* [72] published a review of the literature on the effect of carbohydrate intake on team sport performance, specifically for those studies using soccer, rugby, and field hockey players. Phillips *et al.* [72] concluded that when protocols designed to mimic the demands of team sports are employed with appropriate methodology, the body of research clearly shows a benefit of carbohydrate intake on intermittent high-intensity exercise capacity for soccer, rugby, and field hockey athletes [40,50,56,73,74,75,76]. 

New to this body of literature are two studies examining soccer, rugby, and field hockey [77,78], and two papers specifically with soccer athletes [79,80]. Most of these recent studies further support the previous research which reported ingesting 30–60 g carbohydrate/h improves intermittent high-intensity exercise capacity. For example, compared with placebo, ingestion of a carbohydrate beverage (6.9% solution, 41 g/h) before and during halftime of an intermittent treadmill protocol (*i.e.*, 5 × 15 min bouts demonstrated to mimic the physiologic demands of soccer) resulted in significantly longer time to exhaustion (49% improvement) and distance covered (+1.12 km) during a subsequent run to exhaustion (80% VO_2_peak) [79]. Additionally, in a series of studies with youth athletes using a modified LIST protocol (4 × 15 min followed by an intermittent run to exhaustion), carbohydrate consumption (35 g/h from a 6% solution or 38 g/h from a gel plus water) immediately before and during exercise resulted in longer time to exhaustion (24% and 21% increase, respectively) and distance covered (+157 m and +118 m, respectively) [77,78]. However, in contrast to these results, Goedecke *et al.* [80] reported no benefit of ingestion of a 7% carbohydrate solution (28 g/h) on players’ run time to fatigue after a simulated soccer match using the LIST protocol. The reason for the discrepancy in findings is unclear, but could be due in part to the athletes being tested in a postprandial state (consumed their normal breakfast 2 h prior to testing) and/or the lower rate of carbohydrate intake during exercise in the study by Goedecke *et al.* [80].

Carbohydrate ingestion and intermittent high-intensity exercise capacity has been tested in one basketball study. Welsh *et al.* [56] reported that carbohydrate ingestion (6% solution before and every 15 min during exercise and 18% solution at halftime; overall rate of ~80 g carbohydrate/h) resulted in a 37% longer shuttle run to fatigue at the end of the simulated game. These results are consistent with that of studies with soccer, rugby, and field hockey players, in which a similar 4 × 15 min intermittent high-intensity protocol was used. 

#### Summary

Across a range of intermittent sports, carbohydrate intake (30–80 g/h) during exercise consistently improves intermittent high-intensity exercise capacity; as measured by the duration to fatigue and distance covered in a shuttle run to exhaustion following a simulated game. This finding is perhaps not surprising, given the well-established benefit of carbohydrate intake on endurance exercise capacity [59].

### 5.2. Sprinting

In a 2011 review, Phillips *et al.* [72] concluded that most research with soccer, rugby, and field hockey athletes suggests no benefit of carbohydrate intake on sprint performance [33,40,50,55,81,82,83]. Subsequent work published by Phillips *et al.* [77,78] in adolescent athletes reported no benefit of carbohydrate intake (gel or 6% carbohydrate-electrolyte solution at ~35 g/h) on peak 15 m sprint time or mean 15 m sprint time for each block of the modified LIST protocol. In addition, a recent study by Kingsley *et al.* [47] showed no differences in recreational soccer players’ mean sprint speed during a 90-min soccer match simulation between carbohydrate (54 g/h via a 5.6% solution) and placebo trials. Only two studies have reported enhanced sprint performance with carbohydrate ingestion. In university level soccer players Ali *et al.* [53] reported that ingesting a 6.4% carbohydrate-electrolyte solution (~30 g/h) was associated with a small, but significant 1.2% improvement in mean 15 m sprint performance during the 90-min LIST protocol. In addition, Gant *et al.* [84] reported that cumulative sprint time was improved by 1.2% when soccer players consumed a 6.2% carbohydrate solution (85 g/h) before and during a 60-min LIST protocol. Even so, at present, 10 out of 12 studies have found no significant benefit of carbohydrate ingestion on sprint performance in soccer, rugby and field hockey.

Four studies have examined the impact of carbohydrate intake on sprint performance during a simulated basketball protocol. Baker *et al.* [85] and Dougherty *et al.* [86] employed similar protocols testing the effect of 6% carbohydrate-electrolyte solution (~70–73 g/h) *versus* placebo on adult [85] and youth [86] players’ on-court sprint performance. None of the sprint measures (ladder suicide, 20 court-width sprints, or total sprint time) were impacted by carbohydrate ingestion in adults. However, the youth basketball players experienced significant ~3%–5% improvements in suicide sprint times, mean sprint times, and total sprint times throughout the simulated game with carbohydrate compared with placebo ingestion. In a series of studies, Welsh *et al.* [56] and Winnick *et al.* [87] tested the effects of carbohydrate ingestion on 20 m sprint tests interspersed between 15-min quarters of an intermittent high-intensity shuttle run protocol. Both studies reported significantly faster 20 m sprint times in the fourth quarter of the simulated game with carbohydrate compared with placebo ingestion. Sprint performance improved by ~14% when subjects ingested ~80 g/h of carbohydrate in the study by Welsh *et al.* [56] and by ~3% with ~40 g/h of carbohydrate in the study by Winnick *et al.* [87] In summary, 3 of 4 basketball studies found improvements in sprint performance associated with ingestion of 40–80 g carbohydrate/h before/during simulated games.

One study has tested the effect of carbohydrate intake during a simulated game (50-play scrimmage) on sprint performance in American football players [88]. A 170 mL bolus of 7% carbohydrate beverage or placebo was consumed before warm-up, before the start of the scrimmage, and at 4 separate fluid breaks during the scrimmage (for a total of 71 g carbohydrate). An anaerobic power test, consisting of eight 40-yd sprints separated by a 40-s jog, was conducted before and after the scrimmage. No differences in mean or peak sprint velocity were found between the carbohydrate and placebo conditions [88]. 

#### Summary

To date, the available studies report mixed results regarding the effects of carbohydrate ingestion on sprint performance in protocols designed to mimic the demands of intermittent sports. For instance most studies with a mix of rugby, soccer, and field hockey players suggest no benefit. However, two soccer as well as three basketball studies have found a benefit of carbohydrate ingestion on sprint performance. More research is needed to understand the reason for the disparate results among studies.

### 5.3. Jumping

A few studies have measured the impact of carbohydrate ingestion on vertical jump performance during a simulated basketball model [56,85,86,87]. The jump protocols included maximum vertical jump height [85,86], time to complete a set number of repeated vertical jumps [85,86], and mean height reached during repeated vertical jumps [56,87]. Only one study [87] found a benefit of carbohydrate intake on jump performance; with higher mean jump height during 20 repeated maximum jumps in the fourth quarter of a simulated game achieved with 6% carbohydrate solution (41 g/h) versus placebo. By contrast other published studies reported that maximum vertical jump height [85,86] and repeated sub-maximum vertical jump performance [56,85,86] were not affected by ingestion of a 6% carbohydrate solution (~70–80 g /h) at any point in time during a simulated basketball protocol. 

Vertical jumping is also an important aspect of soccer performance, such as when trying to out jump an opponent to head the ball from a corner kick. However, there are very limited data on the effect of carbohydrate ingestion before/during exercise on jumping performance in soccer. In one study using a reliable maximum jump height test during a soccer heading drill, no significant differences in performance where found between carbohydrate (7.5% carbohydrate solution, 55 g/h) and placebo ingestion during a 90-min soccer simulation [89]. 

#### Summary

The available evidence suggests that carbohydrate intake does not have a consistent benefit on vertical jump performance in basketball players. There are limited data available in other intermittent sports, but one study has also reported no significant beneficial effect of carbohydrate intake on jumping performance in soccer.

### 5.4. Sport-Specific Skill

Dribbling, shooting, passing, and heading are among the types of skills involved in soccer performance. It seems that the most consistent results regarding carbohydrates’ effect on skill performance can be found in the soccer literature as most studies have reported improvements in soccer players’ shooting, dribbling, and/or passing performance with ingestion of a 6%–8% carbohydrate solution at an intake rate of 30–63 g/h [48,53,55,89,90,91]. In fact, Russell and Kingsley [6] recently reported in a systematic review that 6 of 8 studies found that ingestion of 30–60 g of carbohydrate/h (via a 6%–8% solution of glucose, sucrose, or maltodextrin) was associated with an enhancement of at least one aspect of soccer skill performance. For example, Currell *et al.* [89] found a significant ~3%–4% improvement in dribbling and kicking accuracy with 55 g carbohydrate/h ingestion versus placebo. In general, there is a tendency for players’ skill performance to decline during the latter stages of play with placebo intake and it seems that carbohydrate ingestion can attenuate this decline [48,53,55,89]. For example, throughout a 90-min soccer match simulation, professional players’ shot speed decreased by 10.5% with placebo and only 5.2% with carbohydrate ingestion (6% solution, 59 g/h) [48]. Similarly, in a study using the LIST protocol there was a 14% reduction in passing performance from pre- to post-test with placebo ingestion and only a 3% reduction with 52 g carbohydrate/h (via a 6.4% solution) [55]. However, the effect of carbohydrate on passing skill did not reach statistical significance in this study (*p* = 0.07) [55] as well as another study using the same protocol (6% reduction in performance from pre- to post-test with placebo, 1% reduction with 30 g carbohydrate/h; *p* = 0.13) [53]. This perhaps suggests that there is a lot of noise in the measurement of some skills [92,93], making it difficult to detect a significant effect with the relatively small number of athletes tested in most studies. This and other methodological limitations and considerations are discussed in more detail later. Interestingly, soccer is the intermittent sport with the most protocol validation work [32,89,92]; and most of the studies showing some beneficial effect of carbohydrate ingestion on skill performance employed valid and relatively reliable skill tests [48,53,55,89,90]. 

It is important to note, however, that some studies have found no benefit of carbohydrate ingestion on soccer-specific skill. In one study, soccer players experienced no differences in ball shooting performance when they consumed a 6% carbohydrate-electrolyte solution (49 g/h) *vs.* placebo before/during a 90-min soccer simulation protocol [82]. In addition, Zeederberg *et al.* [94] reported no effect of ingesting a 6.9% carbohydrate solution (32 g/h) on players’ proficiency (success rates) in passing, dribbling, heading, and shooting the ball as determined from investigators’ subjective evaluations of video-recorded live matches. 

Basketball-specific skills include shooting, passing, and dribbling. Shooting accuracy is the only skill in which the effect of carbohydrate has been studied to date. Two studies with similar protocols tested the effect of carbohydrate (6% solution, 70–73 g/h) versus placebo ingestion on shooting accuracy during a 4-quarter session of basketball drills/movements to simulate a game. The study in youth players reported improved total shooting accuracy (including “around the world”, 3-point, and free-throw shooting) with carbohydrate ingestion (60% *vs.* 53% with placebo) [86]. By contrast, the study in adults found no effect of carbohydrate on shooting accuracy [85]. Given the similar testing methods and carbohydrate treatments it is difficult to determine the reason for the disparate results between studies. Again, perhaps the inconsistent results are related to a low signal-to-noise ratio associated with the measurement of sport-specific skill. Nonetheless, more work is needed in both youth and adult basketball players to understand how carbohydrate intake might affect shooting accuracy as well as passing and dribbling skills. 

Several studies have tested the impact of a carbohydrate drink on tennis skill (serve and groundstroke performance) as well as overall match performance, with mixed results reported within and among studies [54,95,96,97,98]. For example, one study found improved overall match performance (serve success by 2%–4%), but no change in post-match performance of isolated skill tests when consuming carbohydrate (42 g/h via a 6.4% solution) [54]. Stroke quality for service and defensive rallies during the final stages of a strenuous 2-h training session was improved by ~3%–7% when ingesting a carbohydrate drink (50 g/h) compared with placebo [98]. However, two separate studies reported no impact of carbohydrate intake (34 g/h and 62 g/h via a 6% solution) on match or skill performance [96,97]. In addition, carbohydrate ingestion (46 g/h for women and 61 g/h for men via a 7.6% solution at 15-min intervals and 15.2% solution during one break) had no impact on hitting accuracy during a ball machine test performed after a 4-h tennis match [95]. Many different tests have been used to measure tennis-specific performance, including ball-machine tests, simulated matches against a ball machine or opponent, live matches, or training sessions. The reliability of some of these tests is unknown [54,95]. Nonetheless, there seems to be no consistent pattern regarding the type of test used and the impact of carbohydrate ingestion on skill performance. Thus it is difficult to discern the reason for the mixed results regarding carbohydrate’s effect on tennis skill and match performance. 

Limited information is available on other racquet sports. In a series of studies, Bottoms *et al.* [99,100] tested the effect of 6.3%–6.4% carbohydrate drink ingestion on squash and badminton performance. In squash players, carbohydrate ingestion (43 g/h) had no impact on forehand or backhand straight drive accuracy [100]. In the badminton study, players performed short serve and long serve accuracy tests before and after a 33-min bout of fatiguing badminton-simulated activity. The ingestion of carbohydrate (80 g/h) had no significant effects on badminton short serve or long serve accuracy [99]. 

#### Summary

Carbohydrate intake at a rate of ~30–60 g/h has been associated with a consistent beneficial effect on skill performance in soccer. On the other hand, studies in basketball and racquet sports have found mixed results (at best) from ~35–80 g/h carbohydrate ingestion on sport-specific skills. It is likely that these disparate findings are due, at least in part, to differences in protocol standardization and skill test reliability in soccer compared with basketball and racquet sport studies. Nonetheless, it seems that carbohydrate may be more likely to maintain skill performance when athletes are fatigued (e.g., toward the end of a match). A decline in skill performance typically does not occur unless fatigue is induced and therefore carbohydrate ingestion is not likely to impact performance unless skill is assessed under conditions of fatigue. More work is needed to determine potential effects of carbohydrate ingestion on sport-specific skills in basketball and racquet sports, as well as many other intermittent sports which currently lack any data. 

### 5.5. Change of Direction Speed

Three separate studies have measured the effect of ingesting a carbohydrate solution on change of direction speed in soccer, with conflicting results [80,82,89]. One study that tested athletes in a post-prandial state found no effect of carbohydrate ingestion (28 g/h from a 7% solution) during exercise on players’ performance of a soccer-specific “agility” test [80]. In the other two studies, carbohydrate intake rates were similar (49 *vs.* 55 g/h); however, other methodological differences could account for the disparate findings. The study reporting a significant 2% improvement in change of direction speed with carbohydrate ingestion employed a highly reliable test [89], whereas no reliability data were reported in the study finding a lack of carbohydrate effect [82]. It is also important to note that the complexity of the change in direction tests were different among studies; two involved running and/or dribbling [80,82], while one study involved running only [89]. This is important since the complexity of a performance test could impact its reliability and sensitivity; which in turn, impacts the likelihood of detecting significant differences between carbohydrate and placebo ingestion. In addition, the change in direction tests which incorporated dribbling [80,82] may be a measure of soccer skill, more so than simply change in direction speed *per se*.

Limited data are available on change of direction speed related to basketball, but two studies have reported that the time to complete defensive slide drills during a simulated game were unaffected by ingestion of a 6% carbohydrate solution at an intake rate of ~70–73 g/h [85,86]. A potential limitation, however, is that reliability testing of the drills used in these studies was not conducted [85,86]. 

In one study, a tennis-specific running test was used to measure the effect of ingesting a 7.6% carbohydrate solution (61 g/h by men, 46 g/h by women) on tennis players’ change of direction speed after a 4-h match [95]. There was a small but significant 1.5% improvement in the time to complete the test when players ingested carbohydrate compared with placebo.

One study has tested the effect of carbohydrate ingestion on rugby-specific change of direction speed. Rugby union players performance was unaffected by ingestion of a 9% carbohydrate solution at an intake rate of 110 g/h [83]. The rugby-simulated performance test has been reported to be reliable and sensitive to fatigue [101]. However, it is interesting to note that the carbohydrate intake rate was almost twice that of the upper boundary of the recommended range.

A standardized “hopscotch” test has been used in a few studies to test the effect of carbohydrate ingestion on whole body motor skill and change of direction speed [56,83,87]. In athletes of various team sports, ingestion of a carbohydrate solution (6% and/or 18%, 40–80 g/h) before/during an intermittent high-intensity shuttle running protocol significantly improved motor skill/change of direction speed, particularly during the latter half of the simulated game [56,87]. However, in another study, rugby union players experienced no improvement in the same “hopscotch” test despite consuming 110 g/h of carbohydrate [83].

#### Summary

Similar to findings in the skill literature, the effect of carbohydrate ingestion on change of direction speed in intermittent sport athletes is mixed. However, very limited data on change of direction speed in intermittent sports are available; even more so than that of skill. Additionally, the range of carbohydrate ingestion rates tested is quite varied (~50–110 g/h) making it difficult to compare results across studies. It is also important to note that no studies have employed tests that involve changes in direction in response to a stimulus (agility), which may be a more ecologically valid measurement of intermittent sports performance. For example, while playing defense in basketball, players need to make quick lateral movements in reaction to an opponent’s movements. Similarly, success in tennis is partly dependent upon a player’s ability to get their body in position to return a serve or perform a series of groundstrokes/volleys during a rally. Therefore, research measuring agility, which incorporates movements in reaction to a stimulus, is needed to determine the impact of carbohydrate intake on intermittent sport performance.

### 5.6. Cognition: Attention and Response Time

Attention is one of many aspects of cognition that is critical to basketball performance. For example, success in basketball is dependent upon a player’s ability to concentrate on the coach’s instructions/play call during a time out, avoid distraction from crowd noise when shooting a free throw, and make quick decisions on the court. The effect of carbohydrate ingestion on attention and vigilance-related attention (the ability to maintain attention over a prolonged period of time) [102] has been measured in basketball studies. Ingestion of a carbohydrate drink (6% and/or 18% solution, 40–80 g/h) before/during an intermittent high intensity shuttle running protocol had no impact on athletes’ (including basketball) attention performance (Stroop Color-Word test) [56,87]. Similarly, basketball players’ vigilance-related attention (Test of Variables of Attention) was unaffected by ingestion of carbohydrate (6% solution, 73 g/h) before/during a simulated game [103]. 

One study with squash players reported a significant improvement in choice visual reaction time but no change in auditory reaction time after ingesting carbohydrate compared with placebo [100]. While the tests used in this and the basketball studies (above) are standardized and valid for measuring attention and reaction time they do not involve sport-specific cognitive tasks. Only two studies have included cognitive tasks designed to more closely mimic cognitive demands of specific sports; still no effects of carbohydrate ingestion were found. Tennis specific perceptual skill [97], indicated by a player’s response-accuracy in anticipating the direction of a serve from video clips, was unaffected by carbohydrate ingestion (6% solution, 62 g/h). Another study found no differences in badminton players’ choice reaction time (run 5 m in the required direction according to a light stimulus) between ingestion of drinks containing carbohydrate (6.4% solution, 80 g/h) *vs.* placebo [99]. Although these tests may have been more specific to sport, no data regarding validity or test-retest (*i.e.*, day to day) reliability were reported.

#### Summary

There are limited data available regarding the impact of carbohydrate ingestion on cognitive performance specific to intermittent sport athletes. Results from the studies to date have found very few beneficial effects on attention and response time. However, it is important to keep in mind that there is likely a cognitive component to skill and agility performance. Carbohydrate intake has been shown in some studies to impact sport-specific skills. It is possible that the beneficial impact of carbohydrate ingestion on skill could be mediated in part by its influence on cognition. Nonetheless, more work is needed to elucidate any potential effects of carbohydrate intake on cognitive performance related to intermittent sports. Future studies should include tests that are not only reliable but also relevant to the cognitive demand of sport; such as recalling and executing plays/instructions from a coach and/or making accurate and quick decisions in the face of a dynamic environment typical of intermittent sport competition.

See Table 2 for a summary of findings related to the review of the literature on acute effects of carbohydrate intake on intermittent sports performance.

## 6. Study Limitations, Gaps in the Literature, and Future Directions

Many factors should be considered when interpreting the intermittent sports performance literature. There are many aspects of performance required in team sports and an individual player’s success is dependent upon their ability to coordinate physical movements (sprinting, jumping, and agility) with the simultaneous execution of skills and decision making. Further, performance of skills by an individual player in isolation (e.g., individual skills practice) does not necessarily translate to performance in a competitive game. It is also difficult to mimic the mental stress and complexity associated with game-time performance. These characteristics make it difficult to measure intermittent sports performance in a controlled study. Nevertheless there are limitations (e.g., inability to control confounding factors, as discussed previously) in using live matches to test the effect of carbohydrate ingestion on performance.

**Table 2:** Literature summary: Effect of carbohydrate intake during exercise on intermittent sports performance.

	Sports Tested	Literature Summary	Additional Comments
Intermittent high-intensity exercise capacity	Soccer [73,79,80] Soccer, rugby, and field hockey [40,50,77,78] Basketball [56] Non-specific [74,75,76,81]	Consistent performance enhancement	This finding is perhaps not surprising, given the well-established benefit of carbohydrate intake on endurance exercise capacity
Sprinting	Soccer [53,55,82,84] Soccer, rugby, and field hockey [33,40,47,50,77,78,81,83] Basketball [56,85,86,87] American football [88]	Mixed results	Most studies suggest no benefit of carbohydrate ingestion on sprint performance in soccer, rugby, field hockey, or American football. However, 3 out of 4 basketball studies report a benefit of carbohydrate ingestion on sprint performance in the 4th quarter of a simulated game.
Jumping	Basketball [56,85,86,87] Soccer [89]	Minimal effects	Limited data available.
Skill	Soccer [48,53,55,82,89,90,91,94] Basketball [85,86] Tennis [54,95,96,97,98] Badminton [99] Squash [100]	Mixed results	Consistent beneficial effect of carbohydrate in soccer studies (sport with the most protocol/skill test validation work). Studies in basketball and racquet sports have found mixed results. Skill is difficult to measure and many different tests have been used. More validation work is needed for measurement of skill in court-based and other sports.
Change of direction speed	Soccer [80,82,89,90] Basketball [85,86] Tennis [95] Rugby [83] Non-specific [56,87]	Mixed results	Limited data available for pre-planned change of direction speed. No studies involving changes in direction in response to a stimulus. Difficult to measure. More validation work is needed.
Cognition: Attention and response time	Basketball [56,87,103] Tennis [97] Badminton [99] Squash [100]	Minimal effects	Limited data available. Difficult to measure. Most cognitive tests are not specific to team sports. More validation work is needed.

Another difficultly in interpreting the literature on carbohydrate ingestion and intermittent sport performance is the inconsistent methodologies between studies; some examples include the initial (pre-test) carbohydrate status of the athlete (e.g., fed or fasted; glycogen-depleting exercise or rested the day before testing), the amount and timing of carbohydrate ingested during exercise, the athletes’ level of skill/expertise, and the tests used to measure intermittent sport performance. Many different tests have been used to measure intermittent sport performance and often the reliability and validity of the tests are unknown. Because many of the tests used to measure intermittent sport performance, particularly those for skill, change of direction speed, jumping, and cognition, have inherently high variability (day-to-day reliability, learning effects, *etc.*) this low signal-to-noise ratio may make it difficult to detect significant differences in performance [104]. The paucity of sport protocol and skill/cognitive test validation work represents a significant gap in the intermittent sport literature, particularly for court, batting, and strength and power-based intermittent sports. Another important area missing from intermittent sports research is the dose-response relation between carbohydrate ingestion rate and performance. For future research, it is critical that sport-specific protocols are developed so that the effects of carbohydrate dose-response, as well as the initial carbohydrate status of the athlete and the timing of carbohydrate ingestion, on indicators of intermittent sport performance can be determined. Standardization of the sport-specific protocols and skill tests used would also make it more possible to compare results across studies.

Three main factors should be considered in developing sports-specific performance protocols: validity, reliability, and sensitivity. To be valid, a protocol must resemble the performance that is being simulated as closely as possible. Both the criterion validity (comparison to an objective or criterion measure of performance) and construct validity (can be measured by comparing groups with different abilities) of a protocol are important. For example, studies have reported good construct validity of soccer skills tests. Elite university players had faster mean shot speed and better passing performance than non-elite university soccer players [92]. Similarly, Russell *et al.* [93] reported that professional players performed significantly better than recreational players in at least one outcome measure (speed, precision, or success) for passing, shooting, and dribbling skills. 

Reliability is a measure of the variation (biological and technical) of a protocol and is typically evaluated by determining the test-retest coefficient of variation (CV), standard error of measurement, and/or limits of agreement. For example, in general a CV less than 5% indicates good reliability, whereas protocols with a CV greater than 10% are considered to have relatively poor reliability in sports performance studies [104]. Reliability data have been reported in several soccer studies. For example, shot speed in soccer shooting tests has been reported to have CV’s in the range of ~7%–10% [92,93]. Measures of soccer passing skill performance (e.g., precision, success, performance time) have been shown to be less reliable, with CV’s of ~10%–14% [92,93]. The aspect of soccer performance that seems to have the most varied reliability (CV) results across studies are those that measure shooting performance, including accuracy (2.8%) [89], precision (23.5%) [93], success (14.4%) [93], or points scored (57.8%) [92]. By contrast, dribbling skill seems to be the most reliable performance measure, with CV’s reported to be ~2%–5% for dribbling speed, precision, and success [89,93]. In addition, Ali *et al.* [92] observed more repeatable performance by elite *vs.* non-elite university players in the Loughborough Soccer Passing (e.g., CV’s were 11.2% *vs.* 16.0% respectively, for performance time) and Shooting (e.g., CV’s were 8.4% *vs.* 10.7% respectively for shot speed) tests. These relatively high CV’s (typically >5%–10%), particularly in shooting performance (up to 58%), observed even in elite soccer players, illustrates the inherent variability in skill performance, which can make it difficult to detect statistically significant performance differences in intervention studies. However, because these tests involve decision-making and visual processing, reflecting the dynamic nature of the game rather than technique alone, they have high ecological validity (see [92,105] for more discussion). 

To be sensitive, the protocol must be able to detect small, but meaningful, differences in performance. Sensitivity can be measured using a signal-to-noise ratio, such that the greater the signal or effect (e.g., mean change in performance) and the lower the noise or variability (e.g., within-subject CV) the more sensitive the protocol [104]. Familiarizing subjects with the protocol as well as controlling environmental conditions and extrinsic motivational factors are also important aspects of reliable and sensitive protocols [104]. For more detailed discussions regarding the validity, reliability, and sensitivity of sports performance measures the reader is referred to other reviews [104,105,106,107].

## 7. Recommendations

### 7.1. Amount and Timing of Carbohydrate Intake

Current guidelines for athletes competing in intermittent sports recommend ingesting carbohydrate at a rate of 30–60 g/h during >1 h of training and competition [1,2,3,4] to provide fuel to the muscle and central nervous system [2] to delay fatigue as well as for the potential non-metabolic central effects related to reward and motivation. The findings from the literature review on intermittent sports performance detailed above suggest that 30–60 g/h of carbohydrate intake consistently enhances intermittent high-intensity performance. However, according to the available evidence, studies have shown mixed results from ingestion of 30–60 g carbohydrate/h on sprinting, sport-specific skills, and change of direction speed. Carbohydrate ingestion has minimal effects on jumping and cognitive function (attention and response time) in intermittent sports. It is also important to take into account the fatigue level and carbohydrate status of the individual, since carbohydrate ingestion seems to have the greatest impact on performance under circumstances eliciting permanent fatigue and/or hypoglycemia, such as toward the end of a long match. Thus, carbohydrate intake may be more important for an athlete’s performance when the carbohydrate is consumed before/during a game or high-intensity practice as opposed to before/during a lower-intensity training session.

The exact timing and amount of carbohydrate consumed prior to and during exercise should meet the individual needs and preferences of the athlete, and be determined in relation to such factors as the demands of the sport, training goals, and timing [2]. Although some sports allow frequent ingestion of carbohydrate during a game (e.g., tennis, field hockey, basketball, American Football), other sports limit the opportunity to ingest carbohydrate to infrequent breaks in play and the halftime period (e.g., soccer, rugby). However, Clarke *et al.* [108] showed that ingestion of 45 g of carbohydrate/h (via 1075 mL of a carbohydrate-electrolyte solution) resulted in similar metabolic responses (blood glucose concentration and carbohydrate oxidation), whether the drink was consumed in small volumes at frequent intervals (179 mL at 6 time points, *i.e.*, every 15 min) or in larger boluses (538 mL) before and at halftime of a 90-min soccer specific protocol. These results, as well as a previous study by Clarke *et al.* [109], using a similar design suggest that ingesting carbohydrate in a sports drink before a game and again at half-time is an effective strategy for carbohydrate provision. It is also worth noting, however, that rapid exercise-induced reductions in blood glucose concentrations at the start of the second half of soccer matches have been observed and carbohydrate ingestion does not prevent this response [47,48,49]. At present there is no evidence that this transient glycemia response impacts skill or other aspects of intermittent performance during the early stages of the second half [47,48]. Nonetheless, strategies to attenuate this acute decrease in blood glucose concentration may be an area for future investigation. 

In sports with infrequent opportunities to drink/eat, the ingestion of sufficient quantities of carbohydrate prior to exercise is likely an important strategy to support performance. When the duration of the intermittent sport activity exceeds or may potentially exceed 1 h a pre-exercise meal should be combined with carbohydrate intake during exercise. To our knowledge, no data are currently available directly investigating this practice in intermittent sports. Nevertheless, data from constant pace running exercise suggests that the combination of a pre-exercise meal with appropriate carbohydrate feedings during exercise are superior to maintaining performance compared with when either these interventions are adopted alone [110].

As mentioned previously, the fed/fasting status of athletes varies among studies. Although pre-exercise carbohydrate status represents a potential explanation for some of the mixed results in the literature, there seems to be no consistent pattern of how fed/fasting status impacts the acute effects of carbohydrate supplementation on intermittent sports performance. Beneficial effects of carbohydrate intake on intermittent exercise capacity, sprinting, and change of direction speed have been observed in studies where athletes are fasted (8.5–12 h) [50,56,74,75,79,84,87] as well as studies where athletes are fed a standardized breakfast shortly (2–3 h) [48,54,73,86,89,90,91,95,98,100] before testing. Most studies that measured sport-specific skills fed athletes prior to testing; and the number of studies finding performance-enhancing effects [48,54,86,89,90,91,98] approximated the number of studies finding no effects [85,94,95,96,99,100] of carbohydrate intake during exercise. Two studies by the same group [53,55] have employed glycogen-depleting methods (high-intensity cycling exercise the evening before the trial followed by a low carbohydrate dinner and an overnight fast) prior to testing the acute effects of carbohydrate intake on soccer performance. One study showed significant benefits of carbohydrate ingestion on sprinting and skill (shooting) performance [53], while the other reported no significant effect of carbohydrate on sprinting and skill (passing) performance [55]. Thus, the carbohydrate status of athletes at the start of exercise does not seem to play a major role in dictating the acute effects of carbohydrate intake during exercise.

### 7.2. Form of Carbohydrate

The most common method of providing carbohydrate during intermittent sports is via a liquid solution (e.g., carbohydrate-electrolyte solution). Liquid forms of carbohydrate are a convenient way to replace sweat water and electrolyte losses in addition to meeting fuel needs. The composition of a carbohydrate-electrolyte solution is an important consideration, as the amount and type of carbohydrate impacts the rate of gastric emptying and intestinal absorption of the ingested fluid (see [111] for a more detailed discussion). Most studies in the intermittent sport performance literature have used a 6%–7% carbohydrate solution to facilitate rapid fluid and carbohydrate delivery [84]. Solutions with a higher carbohydrate concentration (e.g., ≥8%) can impair gastric emptying of fluids [112] and increase gastrointestinal distress during intermittent activity [113]. However, it is important to note that not all studies have observed increased gastrointestinal distress with ≥8% carbohydrate solutions [114]. Further, some studies have reported improved intermittent exercise capacity and skill performance associated with carbohydrate ingestion via an 8% solution compared with placebo [91]. It is also noteworthy that solid (bar) or semisolid (gel or chew) carbohydrate is oxidized as effectively as a carbohydrate solution in endurance studies, provided that sufficient water is consumed [115,116]. Carbohydrate gels have also been shown to improve endurance capacity during intermittent high-intensity exercise [73,78]. This is an important consideration for a group of individual athletes which constitute a team. This is because the ability to provide a variety of carbohydrate forms or sources has several implications, including accommodation to individual preferences and intake opportunities as well as adaptation for different fluid intake needs. However, a comparison of oxidation rates or different methods of administering carbohydrate (*i.e.*, solid *versus* liquid) has not been studied in intermittent sports. 

### 7.3. Type of Carbohydrate

In general, carbohydrates can be divided in two categories: those that can be oxidized rapidly (up to 60 g/h) and those that are oxidized at much lower rates (up to about 40 g/h). The faster oxidized carbohydrates include glucose, maltose, sucrose, maltodextrins, and amylopectin starches. The slower oxidized carbohydrates include fructose, galactose, isomaltulose, trehalose, and insoluble (amylose) starches. Exogenous carbohydrate oxidation is mainly limited by the intestinal absorption of carbohydrates. In most studies discussed in this review, the types of carbohydrate consumed before/during intermittent activity are the sugars glucose and sucrose or the glucose polymer maltodextrin. Because these carbohydrates are digested and absorbed at fast rates, they are readily available for rapid oxidation by the muscles (see [117] for review). 

For very prolonged exercise (e.g., ≥2.5 h endurance event) the carbohydrate intake recommendation increases to 90 g/h and should include a blend of multiple transportable carbohydrates (e.g., 2:1 ratio of glucose:fructose) [118]. The glucose transporter (SGLT1) in the intestine becomes saturated at ~60 g/h, while the additional 30 g/h of fructose makes use of the GLUT5 transporter. Utilizing multiple transporters (e.g., the SGLT1 for glucose and GLUT5 for fructose) leads to an increase in the rate of carbohydrate absorption and thus higher carbohydrate oxidation rates (see a review by Jeukendrup [117] for a more detailed discussion). However, the need for multiple transportable carbohydrates may not be as relevant to intermittent sports, given the shorter duration and lower carbohydrate needs compared with endurance sports. Clarke *et al.* [119] found no difference in total carbohydrate oxidation when a glucose or glucose + fructose solution was provided at a rate of 1.0 g/min during a 90 min soccer-specific protocol (total of ~68 g carbohydrate). This finding is perhaps not surprising since saturation of the glucose transporter is not expected to be a limiting factor at this rate of carbohydrate ingestion (60 g/h or 1.0 g/min) [117]. Nonetheless, more work is needed to determine whether there is a benefit of ingesting multiple transportable carbohydrates in intermittent sports. It is currently unclear whether any intermittent sports ever require carbohydrate intake rates higher than 60 g/h. It is possible that sports of long duration and high rates of glycogen utilization could require such high intake rates (e.g., some tennis and soccer matches), but there is currently a lack of direct evidence.

**Table 3:** Considerations for carbohydrate ingestion during intermittent sports.

	Description	Examples
Amount:	30–60 g/h	0.5–1.0 L/h of a 6% carbohydrate solution
Type:	Rapidly digested, absorbed, and oxidized	The sugars glucose, sucrose, and maltose, as well as the glucose polymer maltodextrin and the starch amylopectin
Form:	Liquid, semisolid, or solid	Carbohydrate-electrolyte solution (<8%) or gel, chew, or bar with sufficient water to aid absorption
Other considerations:	Personal preference, experience, goals, and timing	Preference for a certain flavor and/or form of carbohydrate may promote intake and limit gastrointestinal distress

The glycemic index of carbohydrate consumed by athletes, particularly shortly (30–60 min) before exercise, has also been a topic of interest. Consumption of a low glycemic index carbohydrate results in a slow and gradual plasma glucose response whereas a high glycemic index carbohydrate results in a rapid increase in blood glucose concentration followed by a rapid return to baseline values or below (rebound hypoglycemia). Although the glycemic index of a carbohydrate consumed before exercise has significant effects on metabolism, the hypoglycemic effect is transient (lasting ~10 min into exercise) and there is little evidence of any effects on performance. In fact, the large majority of studies have found no differences in endurance performance outcomes (see [63] for review). Fewer studies have been conducted in intermittent sports; but available evidence suggests no significant differences between low and high glycemic index carbohydrates (consumed 2–3 h before exercise) on distance covered in a repeated sprint test during the last 15 min of a 90-min intermittent high-intensity exercise protocol [120,121].

Considerations for the type and form of carbohydrate ingested during training/competition are summarized in Table 3.

## 8. Conclusions

The currently available research suggests there is some potential for carbohydrate ingestion to enhance performance in tests that simulate the intermittent high-intensity nature and skills of intermittent sports. Carbohydrate ingestion consistently improves intermittent high-intensity exercise capacity; however, studies have shown mixed results (improved or no effect) with regards to effects on sprinting, skill, and change of direction speed and minimal effects on jumping and cognition (attention and response time). In most of these studies the amount of carbohydrate consumed was ~30–60 g/h in the form of a 6%–7% carbohydrate solution comprised of sucrose, glucose, and/or maltodextrin. Carbohydrate ingestion seems to have the greatest impact on performance under circumstances eliciting permanent fatigue and/or hypoglycemia. It is also apparent that carbohydrate has more consistent beneficial effects in soccer than other intermittent sports. However, it is unclear whether this is related to the endurance-based nature of soccer, the employment of more rigorously validated soccer protocols, or simply the limited data available in other sports. More research using valid and reliable protocols is needed in other intermittent sports (particularly court, power-based, and batting sports) to determine the potential role of carbohydrate in enhancing performance. In the future, researchers should measure and report the reliability (e.g., test-retest CV), validity (criterion and/or construct), and sensitivity (e.g., signal-to-noise ratio) of the sport-specific protocols and skill tests used to assess performance. In addition, dose-response studies are currently lacking and future work in this area would be helpful in confirming/refining sport-specific carbohydrate intake recommendations. 
